# Advanced Glycation End Products Evolution after Pancreas-Kidney Transplantation: Plasmatic and Cutaneous Assessments

**DOI:** 10.1155/2016/2189582

**Published:** 2016-01-05

**Authors:** La Salete Martins, José C. Oliveira, José Ramón Vizcaíno, Isabel Fonseca, Carlos Gouveia, Donzília Silva, António C. Henriques, Irene L. Noronha, Anabela Rodrigues

**Affiliations:** ^1^Nephrology Department, Hospital Santo António, Centro Hospitalar do Porto, Porto, Portugal; ^2^Unit for Multidisciplinary Research in Biomedicine, Institute of Biomedical Sciences Abel Salazar and University Hospital de Santo António, University of Porto, Porto, Portugal; ^3^Transplantation Department, Hospital Santo António, Centro Hospitalar do Porto, Porto, Portugal; ^4^Clinical Pathology Department, Hospital Santo António, Centro Hospitalar do Porto, Porto, Portugal; ^5^Surgical Pathology Department, Hospital Santo António, Centro Hospitalar do Porto, Porto, Portugal; ^6^Surgery Department, Hospital Santo António, Centro Hospitalar do Porto, Porto, Portugal; ^7^Cellular and Molecular Nephrology Laboratory, Division of Nephrology, University of São Paulo, São Paulo, Brazil

## Abstract

Diabetes mellitus leads to increased Advanced Glycation End Products (AGE) production, which has been associated with secondary diabetic complications. Type 1 diabetic patients undergoing pancreas-kidney transplantation (SPKT) can restore normoglycemia and renal function, eventually decreasing AGE accumulation. We aimed to prospectively study AGE evolution after SPKT. Circulating AGE were assessed in 20 patients, at time 0 (T0), 3 months (T3), 6 months (T6), and 12 months (T12) after successful SPKT. Global AGE and carboxymethyllysine (CML) were analyzed, as well as advanced oxidation protein products (AOPP). Skin biopsies were obtained at T0 and T12. Immunohistochemistry with anti-AGE antibody evaluated skin AGE deposition. AGE mean values were 16.8 ± 6.4 *μ*g/mL at T0; 17.1 ± 3.8 *μ*g/mL at T3; 17.5 ± 5.6 *μ*g/mL at T6; and 16.0 ± 5.2 *μ*g/mL at T12. CML mean values were 0.94 ± 0.36 ng/mL at T0; 1.11 ± 0.48 ng/mL at T3; 0.99 ± 0.42 ng/mL at T6; and 0.78 ± 0.38 ng/mL at T12. AOPP mean values were 130.1 ± 76.8 *μ*Mol/L at T0; 137.3 ± 110.6 *μ*Mol/L at T3; 116.4 ± 51.2 *μ*Mol/L at T6; and 106.4 ± 57.9 *μ*Mol/L at T12. CML variation was significant (*P* = 0.022); AOPP variation was nearly significant (*P* = 0.076). Skin biopsies evolved mostly from a cytoplasmic diffuse to a peripheral interkeratinocytic immunoreaction pattern; in 7 cases, a reduction in AGE immunoreaction intensity was evident at T12. In conclusion, glycoxidation markers decrease, plasmatic and on tissues, may start early after SPKT. Studies with prolonged follow-up may confirm these data.

## 1. Introduction

Patients with diabetes mellitus (DM) have increased production of AGE, the Advanced Glycation End Products [1, 2]. AGE accumulation is only one of the proposed mechanisms for cell and tissue injury in diabetes [3]. There are other possible described mechanisms, such as increased sorbitol formation through the polyol pathway [2, 4], increased protein-kinase C activation [1, 2, 5], and the hexosamine pathway [2]. However, AGE have been the most investigated and may play a central role [1].

AGE are a group of heterogeneous compounds that represent the ultimate product of multiple reactions occurring in several conditions, namely, in the chronic hyperglycemic state of DM. Nonenzymatic glycation begins with interaction and link between the carbonyl group of a reducing sugar and an aminoterminal group of a protein [1, 3]. Complex rearrangements result in early AGE forms, called Amadori products (HbA1c is one of such); progressively they result in more stable AGE precursors (like methylglyoxal) [6] and lately in irreversible long-lasting glycoxidation of the proteins, such as carboxymethyllysine (CML), carboxyethyllysine (CEL), and pentosidine (an AGE with autofluorescent properties) [1, 3, 6]. This process may affect not only proteins (plasma and tissue proteins, such as collagen), but also lipids and nucleic acids [1, 7], then being a measure of overall metabolic and oxidative stress [1, 3]. AGE formation, lipoxidation, and reactive oxygen species (ROS) generation can activate inflammation with consequent tissue damage [3].

Studies did correlate plasmatic and tissue AGE levels to the main micro- and macrovascular complications of DM [1, 3]. AGE formation and deposition have been deeply searched in type 2 (DM2) and type 1 diabetes (DM1), with more recent focus directed to therapeutic possibilities. AGE receptors (RAGE) antagonists [8–11] and other potential targets, possibly preventing AGE formation [1, 2, 7, 9] or promoting AGE degradation and removal [3, 9, 10], are still under investigation.

DM1 patients submitted to simultaneous pancreas-kidney transplantation (SPKT) can restore normoglycemia and renal function, which are two concurrent ways to decrease AGE deposition: reducing AGE formation and increasing their renal elimination. Data on AGE levels after successful SPKT are very scarce. One might aspire that AGE stabilization, or even removal, can be achieved once uremia and hyperglycemia are reverted. However, presuming that it is possible, still the dynamic back-process is not known.

With this study we aimed to collect data on AGE evolution after SPKT. For this purpose, we prospectively measured AGE in the plasma and in skin biopsies, in a group of SPKT patients during the first year after the procedure. The overall protein oxidation has also been assessed, through a test measuring advanced oxidation protein products (AOPP) plasmatic levels.

## 2. Research Design and Methods

### 2.1. Patients

Consecutive patients undergoing SPKT at our center between 23/January/2012 and 6/July/2013, with successful SPKT, who gave their informed consent were enrolled in this study (only one patient excluded, due to pancreas graft thrombosis). Twenty SPKT patients were included for measurement of plasmatic AGE levels; in 15 of these, skin biopsies were obtained to perform the histological and immunohistochemistry analysis of epidermal and dermal AGE deposition.

SPKT was performed with systemic-enteric diversion. Immunosuppression comprised antithymocyte globulin, tacrolimus, mycophenolate, and steroids. Steroid withdrawal after the sixth month is a general practice in our unit, if immunological events are not observed. At last follow-up, steroids were totally withdrawn in 30% of the patients; all the others, except one, were taking ≤5 mg/day of prednisone.

### 2.2. Sample Collection

AGE were prospectively analyzed in skin deposits and in plasma in these SPKT, from time 0 (the day of the transplant, or T0) to 12 months (T12) after the procedure. T0 values (date of transplantation) obtained for each studied marker were considered their basal (reference) levels.

Blood samples were collected in evacuated tubes without additive at T0 and thereafter at 3 months (T3), 6 months (T6), and T12 after the transplant. The first skin biopsy was obtained at T0, during the kidney transplantation surgery; the second one was obtained through a 5 mm skin punch at T12, from the left abdominal wall, 2-3 cm below the scar of the surgical incision used to perform the kidney transplantation. The lower abdominal wall is a part of the body with low chronic UV-exposure and the local of the two biopsies was very close.

Samples (blood and tissue) collection was delayed at least one week for T3 samples; two weeks for T6 samples; or 2–4 weeks for T12 samples, whenever there was an infection and/or transient mild graft dysfunction.

Besides AGE evaluation, in the 4 blood samples collected from each patient, we also analyzed fasting blood glucose, glycated hemoglobin (HbA1c), total cholesterol, triglycerides, low-density lipoprotein-cholesterol (LDL-c), high-density lipoprotein-cholesterol (HDL-c), and C-reactive protein (CRP). Additionally, 24-hour urinary protein excretion was measured on T12, and estimated-glomerular filtration rate (e-GFR) was calculated based on MDRD equation. Blood pressure was recorded in each visit. Hypertension (>130/85 mmHg), hypertriglyceridemia (>150 mg/dL), hypercholesterolemia (>200 mg/dL), high LDL-c (>130 mg/dL), and low HDL-c (<40 in men, <50 mg/dL in women) were defined according to the National Cholesterol Educational Program (NCEP/ATPIII) criteria for metabolic syndrome.

Skin samples from healthy subjects have been provided by the Pathology Department of Santo Antonio Hospital, Porto, from its archive. These were obtained from margins of biopsies made to analyze skin lesions of the dorsal or abdominal wall, which were of benign origin (nevus). Healthy skin from 6 nondiabetic subjects aging between 30 and 45 years was then used as control samples, to assess AGE deposition in the absence of diabetes and within this age range.

### 2.3. Biochemical Studies

Blood samples were centrifuged without delay and the serum was aliquoted and stored frozen at −80°C, until analysis.

Global plasmatic Advanced Glycation End Products (AGE) were evaluated using a competitive ELISA-Kit, OxiSelect AGE (STA-817, Cell Biolabs, Inc., San Diego, CA). N^Є^–(Carboxymethyl) lysine (CML), a specific AGE, was evaluated using a competitive ELISA-Kit, OxiSelect CML (STA-816, Cell Biolabs, Inc., San Diego, CA). Oxidative state was evaluated with a colorimetric kit for the detection of Advanced Oxidation Protein Products, OxiSelect AOPP (STA-318, Cell Biolabs, Inc., San Diego, CA). Biochemical analyses were performed according to manufacturer's indications for each assay.

### 2.4. Skin Histological/Immunohistochemistry Studies

After excision, skin samples were immediately fixed in 10% neutral buffered formalin for 24 h and embedded in paraffin wax. Serial 3 *μ*m cuts were obtained from each block. These sections were stained with hematoxylin-eosin and others used to analyze AGE deposition, through immunohistochemistry assay. After deparaffinization and rehydration, these sections were incubated with the primary polyclonal IgG antibody anti-AGE (ab23722, Abcam, Cambridge, UK) diluted 1 : 5000, for 20 minutes at room temperature, according to the manufacturer's instructions. Immunohistochemistry protocol from Ventana Benchmark Ultra (Ventana Medical Systems, Inc., Roche) has been followed, using detection system Optiview DAB, IHC Detection Kit. Sections were counterstained with hematoxylin and then dehydrated and coverslipped under DPX mountant.

A semiquantitative AGE assessment was made based on its immunoreaction intensity and graded on a scale from 0 (absent) to 1+ (weakly positive), 2+ (moderately positive), or 3+ (strongly positive). All the samples were analyzed by the same pathologist, in two different occasions, and this was a blind analysis: characteristics of patients and the timing of biopsy were not known by the pathologist before observation.

### 2.5. Statistical Analysis

Variables distribution was studied by Kolmogorov-Smirnov test. Results are presented as mean ± standard deviation for continuous, normally distributed variables, or as medians and 95% confidence interval for nonnormal distribution variables (e.g., AOPP). Percentages were used for categorical data. A repeated measures ANOVA was used to compare AOPP, AGE, and CML between time points. Multiple comparisons were adjusted using Bonferroni's test. The effect of potential confounding variables, such as age, gender, previous time on dialysis, diabetes time, dyslipidemia, HbA1c, and creatinine clearance, was then analyzed on longitudinal changes of the three markers also using repeated measures ANOVA.

Statistical analysis was performed using SPSS software version 22.0 (SPSS, Chicago, IL, USA) and *P* < 0.05 was considered statistically significant.

## 3. Results

### 3.1. Demographic and Clinical Patients' Characteristics

Baseline and post-SPKT patients' characteristics are presented in Table 1. Their age at transplantation date ranged from 28 to 47 (mean 36.7) years; their time on dialysis from 2 to 40 (mean 18) months; and their diabetes evolution time from 17 to 33 (mean 26) years. Excessive weight was not observed in this sample of DM1 patients; 20% were active smokers before SPKT; very poor glycemic control was evident in 35%, who presented HbA1c ≥9% (≥74.9 mmol/mol). Three patients needed reoperation (due to infection, bleeding, and partial pancreatic venous thrombosis, one each) and 3 had an early acute rejection, efficiently treated. All of these patients have kept both grafts functioning during the study follow-up.

After SPKT, graft function remained stable. The rate of actively smoking patients has decreased (5%). Hyperlipidemia and hypertension prevalence was low: the percentage of patients taking antihypertensive medication or statins was 15% and 5%, respectively. Mean BMI before and after SPKT was similar, although we have noted weight gain in 9 and weight loss in other 8 patients. Two patients had a BMI >25 kg/m^2^ at T12. These patients had stable graft function and favorable lipid profile evolutions after SPKT (shown in Table 2).

### 3.2. AGE, CML, and AOPP Plasmatic Levels after SPKT

**Figure 1:**
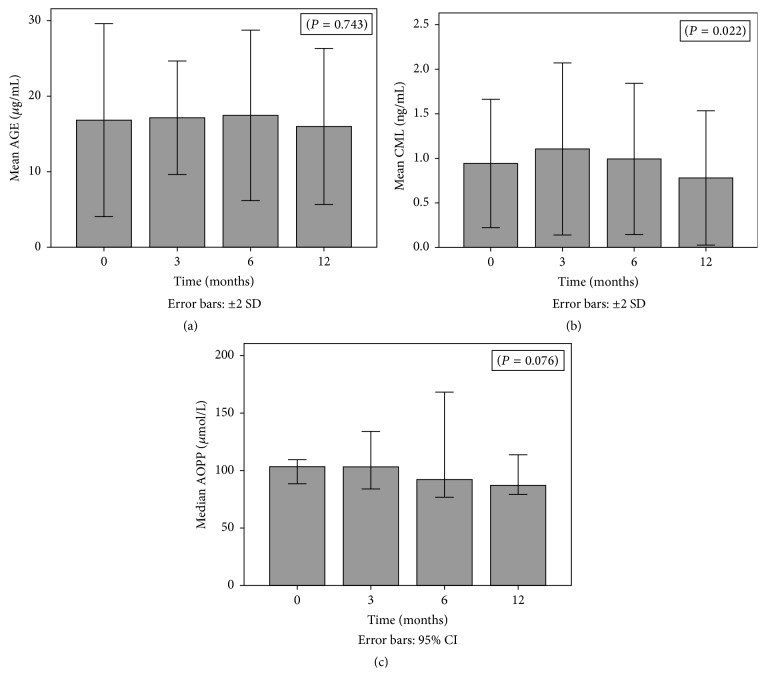
(a) AGE variation from time 0 (T0) to time 12 (T12). (b) CML variation from time 0 (T0) to time 12 (T12). (c) AOPP variation from time 0 (T0) to time 12 (T12).

AGE, CML, and AOPP results during the first year, at different 4 time points, are represented in Figures 1(a), 1(b), and 1(c), respectively.

An increase in the mean values of AGE, CML, and AOPP was registered from T0 to T3. AGE levels have also increased from T3 to T6, a fact not observed for CML and AOPP, for which the decrease started after the 3rd month. From T6 to T12, all the 3 markers have decreased, reaching levels below those registered before transplantation. AGE mean values were 16.8 ± 6.4 *μ*g/mL at T0; 17.1 ± 3.8 *μ*g/mL at T3; 17.5 ± 5.6 *μ*g/mL at T6; and 16.0 ± 5.2 *μ*g/mL at T12 measurements. These variations did not reach statistical significance. CML mean values were 0.94 ± 0.36 ng/mL at T0; 1.11 ± 0.48 ng/mL at T3; 0.99 ± 0.42 ng/mL at T6; and 0.78 ± 0.38 ng/mL at T12 measurements. The observed variation from T0 to T12 was statistically significant (*P* = 0.022). AOPP mean values were 130.1 ± 76.8 *μ*Mol/L at T0; 137.3 ± 110.6 *μ*Mol/L at T3; 116.4 ± 51.2 *μ*Mol/L at T6; and 106.4 ± 57.9 *μ*Mol/L at T12 measurements. AOPP variation was almost statistically significant (*P* = 0.076).

Diabetes duration and age at transplantation date did not significantly correlate with T0 and T12 AGE, CML, or AOPP levels. Time on dialysis was the single factor with nearly significant positive correlation with CML values (*P* = 0.071). Poor glycemic control before transplantation (fasting glucose and HbA1c), as well as hypertension, also did not influence the values of these 3 markers. Additionally, we could not find any association between T12 HbA1c and T12 values of the 3 markers. The number of patients with active smoking (*n* = 1), not taking aspirin (*n* = 1), taking ACEI (*n* = 3), or with any marker of dyslipidemia (*n* = 4) on T12 was too small to study their correlation with AGE, CML, or AOPP at T12.

### 3.3. AGE Skin Deposits from T0 to T12 after SPKT

On histological skin examination we verified that the AGE immunostaining was invariably negative in some specific cells/areas: the outer epidermal layer (stratum corneum), the erector pili muscle, and the eccrine sweat glands. In other cells/areas, immunoreaction for AGE was invariably positive, such as fat cells, vascular endothelial cells, dermal collagen fibers (on superficial dermis 2+/3+, on deeper dermis 3+), and perivascular collagen. The other layers of the epidermis (granular, spinous, and basal) and the hair follicle presented several distinct AGE immunostain patterns and intensity. Hair follicle layers, whenever hair follicle was present in the section, normally follow the same pattern and the intensity of the epidermal layers immunoreaction.

**Figure 2:**
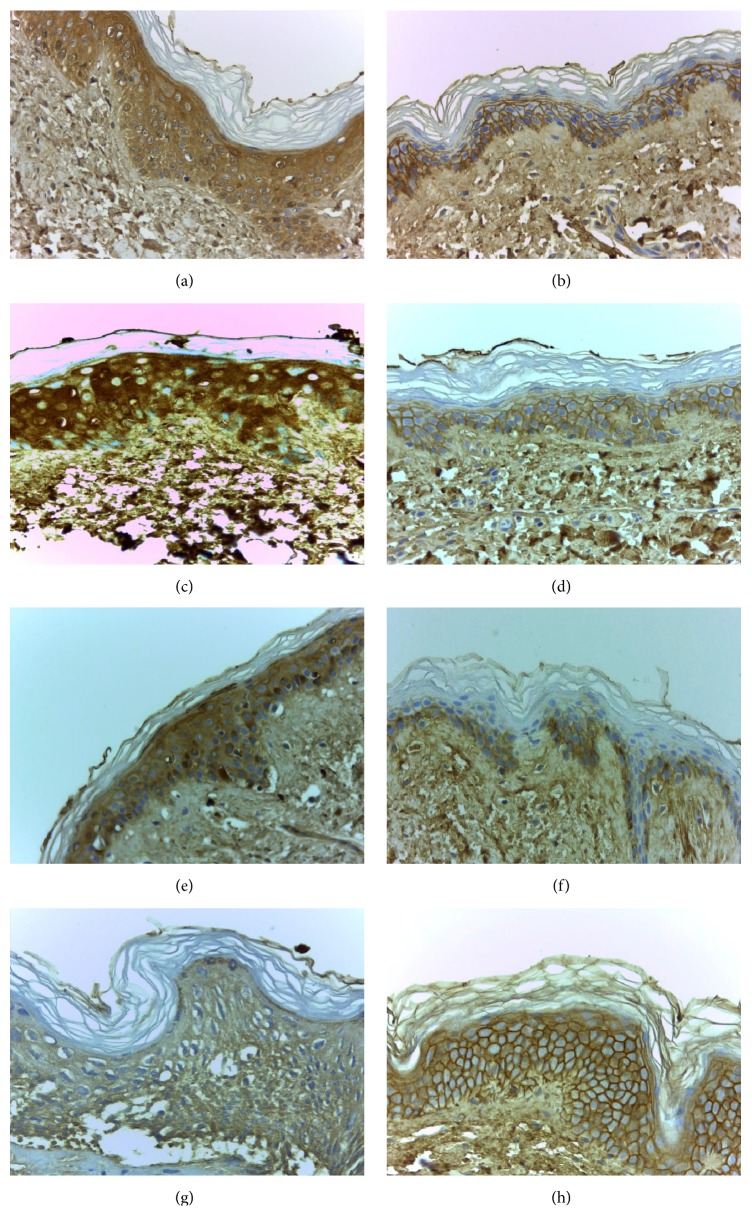
Epidermal immunostaining for AGE:* patient 4* before (a) and after SKPT (b);* patient 8* before (c) and after SKPT (d);* patient 10* before (e) and after SKPT (f);* patient 11* before (g) and after SKPT (h) (400x amplified, hematoxylin counterstained). Images showing the main immunostaining changes, from a diffuse cytoplasmic to an interkeratinocytic or peripheral pattern, often less intense, at time of 12 months.

**Figure 3:**
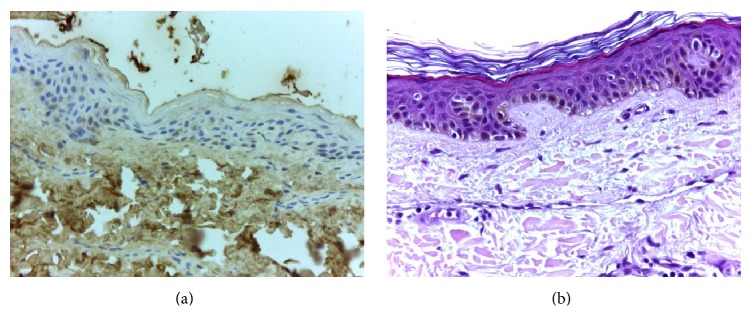
Image (a) represents a negative control for epidermal AGE immunostaining, from a young healthy individual. Irrelevant changes were found on hematoxylin-eosin staining in our patient population (exemplified on image (b)).

Table 3 explains the specific sites with positive immunoreaction for AGE and the respective intensity. The most common finding, observed in 11 among the 15 cases, was a change from a cytoplasmic diffuse immunoreaction pattern on T0 to an interkeratinocytic pattern on T12, saving the central cytoplasmic area and only peripherally staining the cells, with an aspect usually described as “chicken wire” pattern. At least in 7 cases, we have also observed a decrease in the intensity of AGE immunoreaction one year after SPKT (from 3+ to 1+, or from 2+ to 1+). To illustrate these changes we present 4 cases in Figure 2, which exemplify the modifications observed from pretransplant to one year later. On hematoxylin-eosin staining, no relevant changes were found in our study population; and, in young healthy controls, epidermal AGE immunostaining was negative (as illustrated in Figure 3).

## 4. Discussion

Several studies did confirm the association between AGE accumulation and diabetic microvascular complications [1–3, 6, 9], namely, retinopathy [12, 13], neuropathy [7, 14], and nephropathy [15, 16], and also macrovascular disease, such as cardiovascular (CV) [9, 17, 18] and peripheral artery disease [9, 19]. Additionally, it seems that AGE can be directly toxic to pancreatic beta-cells [6, 11]. Exogenous sources of AGE, from diet or smoking, are other contributors to their imbalance and accumulation [1, 3, 6]. AGE formation is not an exclusive mechanism of diabetes. Many other diseases may induce AGE overexpression, such as renal diseases evolving to renal failure [1, 8], neoplasms [8], Alzheimer's disease [1, 7, 8], arthritis [1, 8], and CV disease itself [1, 11]. Furthermore, even unspecific inflammation and aging promote AGE production [1, 7]. Since AGE depend on renal function for their excretion, chronic renal insufficiency also leads to AGE accumulation [1].

RAGE are activated by increased AGE exposure; they respond with overexpression and contribute to ROS formation and inflammation [1, 6, 8]. There are several AGE receptors, some of them with protective antioxidant effects, working to control excessive oxidative stress, whereas others, like RAGE, have prooxidant properties [6]. The search for efficient RAGE blockers is still ongoing.

SPKT treats two diseases, DM1 and end-stage renal disease, and is performed in young patients (most under 50 years of age). Therefore, this is certainly an interesting group of patients to study AGE evolution. Results from AGE levels are very difficult to interpret and there are not standardized methods of detection. Moreover, it remains unclear which AGE should be measured and where to obtain more reliable results, whether in plasma or in tissues [9, 20]. Plasma levels reflect AGE linked to proteins with higher turnover rate (circulating proteins); tissue levels probably reflect better those AGE linked to low turnover proteins such as collagen and, consequently, the tissue damage [20]. For this reason, stabilization or improvement of diabetic secondary complications, thought to be associated with AGE formation and deposition, may eventually occur lately after SPKT [20]. This is the reversal face of the “metabolic memory” phenomenon observed in diabetic patients, a concept that came from the Diabetes Control and Complications Trial-Epidemiology of Diabetes Interventions and Complications (DCCT-EDIC) research: several studies demonstrated a slower progression of diabetic complications in the group of patients who have received intensive insulin treatment, a persistent benefit more than 10 years after the end of the treatment [21–23]. Established tissue lesions certainly are not easily and rapidly reverted, even under maintained normal glycemia and renal function, after successful SPKT [20].

The few studies in transplanted patients [24, 25], one of them comparing a small number of SPKT to kidney alone transplants, have not been able to demonstrate additional benefits with the pancreas graft and euglycemia, besides the correction of renal failure with a kidney transplant. The authors could find a decrease in pentosidine plasma levels in kidney and pancreas-kidney transplants, but not in tissue pentosidine levels [25].

AGE measurement can in fact be made in plasma [26–30], in urine [26], or in tissues, skin being the most often used tissue [31, 32]. Among several compounds already studied, CML is the best characterized AGE [29] and the most consistently assessed one in plasma analysis [26–30]. Higher plasmatic CML levels correlated with higher thickening rate of the glomerular basement membrane [26], increased arterial stiffness [27], increased coronary artery calcification [28], and higher incidence of fatal and nonfatal CV events [30] in diabetic patients; they even correlate with CV events in elderly nondiabetics subjects [33]. In studies performed in chronic kidney disease patients, AOPP plasmatic levels have also been associated with atherosclerotic events in the predialysis stage [34]. Additionally, it was demonstrated that these levels increase after dialysis [35] and AOPP have been proposed as a reliable marker of oxidant-mediated protein damage. AGE accumulation may be directly assessed in tissues, by immunohistochemistry methods [31, 32, 36], or extracted through acid hydrolysis and enzymatic digestion and then measured by biochemical assays [37]. High cutaneous AGE expression has been correlated with skin damage due to sun exposure [31] in nondiabetic patients. In diabetic patients, it has been correlated with dermal inflammation and denervation [32] and with faster progression of microvascular [37] and macrovascular [36] disease.

These were the main reasons why, in our study, we decided to use the assays explained above. One assay was chosen to assess global plasma AGE levels, another one to specifically assess CML levels, and AOPP assay to evaluate protein oxidation. With these 3 markers we can evaluate the overall oxidative status in these patients. Skin deposits were determined by immunohistochemistry, a manner to evaluate tissue lesion and its progression after SPKT.

SPKT patients in our center are strongly encouraged to abolish smoking and to avoid nonhealthy food, external sources of AGE. Smoking habits in these transplanted patients were very rare: only 1 out of 20 remained as an active smoker. Given that, the possible interference of smoking in AGE results in our study is very unlikely. The same assumption can be made regarding inflammation: CRP was almost always steadily low after the first months, data shown only for time of 12 months.

Our study group presented stable pancreas and kidney graft function. CV risk factors, such as hypertension and hyperlipidemia, were generally well controlled. Hypertension frequency decreased after SPKT, from 65% to 30%. Only 10% presented hypertriglyceridemia and 5% hypercholesterolemia after SPKT. The rate of patients needing statins and ACEI was low. Statins [20] and ACEI have been proposed as potential preventers of AGE formation and accumulation [1, 9, 10], as well as aspirin [1, 7]. Per protocol, all of our SPKT are under aspirin after discharge. In this study group, all the patients but one were under aspirin. This homogeneity does not allow us to confirm or to exclude the contribution of these drugs (ACEI, statins, and aspirin) for the results. Still, based on all these facts, we have assumed that the changes observed in our study, regarding AGE, CML, and AOPP levels, may be attributed mainly to normoglycemia restoration and to renal function normalization.

What we observed was a transient increase in AGE, CML, and AOPP after SPKT, instead of an immediate decrease. However during this initial period after SPKT there are several well-known inflammatory/infectious insults, or even high-doses of new drugs, such as immunosuppressors, that may explain the initial increment of these markers. Major surgery, indwelled catheters, episodes of wound, and urinary, abdominal, or systemic infections, among other possible complications, all may contribute to an initial inflammatory state in SPKT patients. Inflammation usually leads to an increase in the oxidative processes. The decrease of the oxidative markers after the 3rd or after the 6th month, although statistically significant only for CML, has been an interesting finding. Once both the rapidity or the reversibility of glycoxidation and protein oxidation processes are not known in the short term after SPKT, we cannot say these were expected results; yet these were not totally surprising results. The limited sample size may also explain the lack of significance of markers' variation.

The same interpretation can be made for skin results. Changes observed from T0 to T12 are in accordance with a reduction in epidermal AGE deposits. The dermal tissue lifetime is considerably longer than that of epidermal tissue. Our observation that the dermal fat cells and collagen fibers were invariably positive for AGE, irrespective of the timing of the biopsy, is consistent with this fact. For this reason our attention was focused on epidermal layers, with higher turnover. In the majority of patients we have observed a modification from an initial diffuse cytoplasmic immunoreaction to an immunoreaction only at the periphery of the cells one year later. Besides this change in pattern, intensity has also decreased, clearly in 7 out of 15. Patient 12 did not present skin AGE deposition, as seen in Table 3. Immunochemistry was repeated and negativity confirmed. Among the other patients included, this patient had the second shortest time interval between diabetes diagnosis and SPKT. Perhaps more importantly, he had an insulin pump and a very good glycemic control: he presented the lowest HbA1c level (6.1%, as shown in Table 2) within our study population, at time of SPKT. It is certainly forced to make definitive conclusions based on data obtained from a single patient. However, the most reasonable explanation is that strict glycemic control protects diabetic patients from the accelerated glycosylation process. There are inherent limitations of this semiquantitative assessment from 0 to 3+; however, this is currently the most often used method to subjectively quantify the immunoreaction intensity in immunohistochemistry.

Certainly, it will be of interest to extend the follow-up of these patients and AGE measurements, in order to analyze their progressive evolution after SPKT, in those maintaining both grafts functioning. Reminding of the knowledge that emerged from several studies, which led to the “metabolic memory” concept, it is very unlikely that AGE reduction within a short time period after uremia and hyperglycemia reversion can produce significant and measurable cell injury improvements, such as relevant changes in neuropathy or vasculopathy parameters, traducing microvascular disease improvements. In the mid- and long-term we hope these improvements will be apparent and quantifiable.

C-reactive protein was the single inflammation marker analysed in this study, due to economic constraints. However, we are aware of other inflammatory factors potentially interfering with AGE production. Another future point of interest will be to find if AGE evolution will eventually correlate with other markers of inflammation. For instance, it is our aim to proceed to further studies, including markers such as inflammatory cytokines (IL-6, among others), as well as markers of vascular cell apoptosis and platelet activation.

Skin autofluorescence (SAF) measure is a promising noninvasive method to evaluate AGE deposition which correlated with AGE levels determined by biochemical analysis of skin biopsies [38]. In uremic patients under dialysis, data obtained on AGE levels through the AGE-Reader were associated with CV mortality [39]. Even in early stages of chronic renal disease, several studies could find a correlation between SAF and CV disease [20]. Additionally, in diabetic patients, data from SAF could be associated with vascular damage [40]. SAF reading needs, however, some adjustments that can affect the accuracy of the method. There are no standardized units; it has to be corrected to ethnic, age, and skin phototype characteristics, and it should be measured in the same part of the body in consecutive measurements, to avoid biases from different UV-exposure zones. Even so, when widely available and taking into account the necessary adjustments, this may be a practical method to measure AGE accumulation, also in SPKT patients.

We have not been able to find any factors clearly associated with the variation of AGE, CML, and AOPP levels in our group of patients.

Based on our results, we can conclude that skin and plasmatic glycoxidation markers, in DM1 patients, may in fact start to decrease during the first year after SPKT. Further studies in a larger sample and with extended follow-up are needed to confirm these results.

## Figures and Tables

**Table 1:** Patients' demographic and clinical characteristics.

	Total group (*n* = 20)
*Before SPKT*	
Age (years)	36.7 ± 5.4
Female gender	11 (55%)
Time of diabetes (years)	26.0 ± 5.3
Time on dialysis (months)	18 ± 11
HbA1c (%)	8.29 ± 1.61
(HbA1c – mmol/mol)	67.1 ± 17.1
HbA1c ≥9% (≥74.9 mmol/mol)	7 (35%)
Fasting glucose (mg/dL)	304 ± 129
Active smoking (*n*/%)	4 (20%)
Body mass index (BMI) (kg/m^2^)	22.4 ± 2.6
BMI >25 kg/m^2^ (*n*/%)	0 (0%)
Hypertension (>130/85 mmHg) (*n*/%)	13 (65%)
*After SPKT (12 months)*	
HbA1c (%)	5.34 ± 0.31
(HbA1c – mmol/mol)	34.9 ± 3.4
HbA1c ≥6% (≥42.1 mmol/mol)	0 (0%)
Fasting glucose (mg/dL)	83 ± 9
e-GFR^*∗*^ (mL/min/1.73 m^2^)	77.5 ± 15.5
Urinary protein excretion (g/24 h)	0.071 ± 0.093
Active smoking (*n*/%)	1 (5%)
BMI (kg/m^2^)	22.4 ± 2.5
BMI >25 kg/m^2^ (*n*/%)	2 (10%)
Taking aspirin (*n*/%)	19 (95%)
Hypercholesterolemia (>200 mg/dL) (*n*/%)	1 (5%)
Hypertriglyceridemia (>150 mg/dL) (*n*/%)	2 (10%)
Low HDL-c^*∗∗*^ (*n*/%)	2 (10%)
High LDL-c (*n*/%)	0 (0%)
Taking statins (*n*/%)	1 (5%)
Hypertension (*n*/%)	6 (30%)
Taking ACEI (*n*/%)	3 (15%)
C-reactive protein (CRP) (mg/L)	1.58 ± 1.05
CRP >5 (mg/L)	0 (0%)

^*∗*^e-GFR: estimated glomerular filtration rate (MDRD calculation).

^*∗∗*^Low HDL-cholesterol defined as <40 mg/dL in men and <50 mg/dL in women.

ACEI: angiotensin converting enzyme inhibitors.

**Table 2:** Patients' graft function and lipid profile evolution during the first year.

Pts	Creat T3	Creat T6	Creat T12	GFR T3	GFR T6	GFR T12	Gluc T0	Gluc T3	Gluc T6	Gluc T12	HbA1c T0	HbA1c T3	HbA1c T6	HbA1c T12	Chol T0	Chol T3	Chol T6	Chol T12	TG T0	TG T3	TG T6	TG T12	LDL-c T12	HDL-c T12	CRP T12
*n* = 1	0.95	1.00	1.17	71	67	64	469	78	72	75	10/85.8	5.5/36.6	5.3/34.4	5.1/32.2	136	212	158	170	229	84	130	91	95	55	1.4
*n* = 2	1.33	1.24	1.18	65	69	72	268	78	89	81	6.8/50.8	5.6/37.7	5.4/35.5	5.4/35.5	161	141	156	176	100	91	81	67	86	75	1.6
*n* = 3	0.92	0.85	0.96	103	107	104	246	76	82	79	7.4/57.4	5.4/35.5	5.2/33.3	4.9/30.1	186	110	112	147	203	78	82	59	84	61	1.2
*n* = 4	1.40	1.35	1.45	59	61	58	169	87	85	85	6.5/47.5	5.3/34.4	5.2/33.3	5.1/32.2	97	126	151	122	77	108	83	98	58	57	2.6
*n* = 5	1.05	1.02	0.93	88	91	95	315	91	90	88	8.5/69.4	5.4/35.5	5.3/34.4	5.1/32.2	80	101	113	97	96	39	27	20	16	67	3.3
*n* = 6	1.09	1.04	1.02	64	67	68	345	72	84	79	11/96.7	5.3/34.4	5.6/37.7	5.3/34.4	230	226	212	207	155	99	76	84	115	58	2.1
*n* = 7	0.75	0.81	0.85	88	85	83	217	69	71	64	9.8/83.6	6.1/43.2	5.5/36.6	5.5/36.6	168	147	139	155	151	127	88	59	70	70	1
*n* = 8	1.27	1.05	1.30	58	66	57	482	88	77	90	7.3/56.3	5.8/39.9	6.0/42.1	5.9/41.0	190	188	177	158	140	78	98	76	79	64	2.5
*n* = 9	1.13	0.95	0.93	59	71	74	110	86	82	81	6.7/49.7	5.4/35.5	5.2/33.3	5.2/33.3	192	213	271	192	263	255	245	158	120	40	1.2
*n* = 10	0.84	1.01	0.90	89	76	87	162	79	81	73	10/85.8	4.9/30.1	5.4/35.5	5.3/34.4	139	183	202	172	191	229	146	96	86	76	1.3
*n* = 11	0.91	1.01	0.95	81	72	77	189	77	71	88	8.2/66.1	5.6/37.7	5.1/32.2	5.2/33.3	171	176	183	181	141	106	89	65	111	65	0.7
*n* = 12	0.75	0.80	0.65	91	88	110	277	90	86	84	6.1/43.2	6.0/42.1	5.7/38.8	5.4/35.5	146	215	193	188	104	118	97	72	99	67	1.5
*n* = 13	1.90	1.80	1.36	36	39	51	523	100	104	96	7.2/55.2	6.3/45.4	6.2/44.3	5.9/41.0	190	188	185	189	322	265	131	136	94	57	0.9
*n* = 14	1.37	1.39	1.29	66	65	70	116	83	80	69	8.0/63.9	5.4/35.5	5.2/33.3	5.0/31.1	195	186	179	160	106	107	99	69	71	79	0.7
*n* = 15	1.27	1.24	1.20	70	73	76	487	91	93	92	9.3/78.1	6.2/44.3	5.9/41.0	5.7/38.8	171	117	122	148	100	96	92	87	76	55	0.7
*n* = 16	1.38	1.30	1.30	64	69	69	405	98	86	87	6.7/49.7	6.4/46.4	6.3/45.4	5.8/39.9	152	149	144	137	113	106	95	91	59	60	1.1
*n* = 17	0.85	0.88	1.09	87	85	82	279	84	75	101	9.7/82.5	6.1/43.2	5.5/36.6	5.6/37.7	181	129	137	139	183	56	62	66	77	52	0.7
*n* = 18	0.86	1.09	0.99	110	95	97	405	79	100	91	11/96.7	6.4/46.4	6.2/44.3	5.1/32.2	138	188	221	161	79	136	145	149	100	24	1.5
*n* = 19	0.90	0.89	0.95	79	79	76	212	63	72	75	6.6/48.6	5.2/33.3	5.3/34.4	5.0/31.1	151	225	175	193	107	111	115	74	123	55	0.7
*n* = 20	1.13	1.25	1.16	84	77	79	398	100	86	88	8.6/70.5	5.7/38.8	5.4/35.5	5.3/34.4	134	202	196	188	134	172	168	156	113	52	4.8

	Mean	Mean	Mean	Mean	Mean	Mean	Mean	Mean	Mean	Mean	Mean	Mean	Mean	Mean	Mean	Mean	Mean	Mean	Mean	Mean	Mean	Mean	Mean	Mean	Mean
	1.10	1.10	1.08	76	75	78	304	83	83	83	8.3/67.2	5.7/38.8	5.6/37.7	5.3/34.4	160	171	171	164	150	123	107	89	87	59	1.6

	SD	SD	SD	SD	SD	SD	SD	SD	SD	SD	SD	SD	SD	SD	SD	SD	SD	SD	SD	SD	SD	SD	SD	SD	SD
	0.29	0.24	0.20	18	14	16	129	10	9	9	1.6/17.5	0.44/4.6	0.38/4.2	0.30/3.3	35	40	40	27	65	62	46	36	25	13	1.0

Creat: serum creatinine (mg/dL); GFR: e-GFR (mL/min/1.73 m^2^, MDRD calculation); Gluc: fasting glucose (mg/dL); HbA1c: glycated hemoglobin (%/mmol/mol); Chol T: total cholesterol (mg/dL); TG: triglycerides (mg/dL); LDL-c: low-density lipoprotein-cholesterol (mg/dL); HDL-c: high-density lipoprotein-cholesterol (mg/dL); CRP: C-reactive protein (mg/L); Pts: patients; SD: standard deviation; T0: time 0; T3: 3 months; T6: 6 months; T12: 12 months.

**Table 3:** Skin biopsies: AGE immunoreaction pattern and intensity before and after SPKT.

Case number (patients)	SPKT	Epidermis	Epidermis	Epidermis
		AGE immunoreaction	Immunoreaction pattern	Intensity
		(layers with positive immunostain)	(peripheral; diffuse; Mixt: both coexist)	(from 0 to 3+)
1	Before	Basal, spinous, granular	Diffuse cytoplasmic	2+ (basal layer 1+)
	After	Basal, spinous, granular	Peripheral/interkeratinocytic	2+

2	Before	Basal, spinous	Peripheral/interkeratinocytic	1+
	After	Basal	Peripheral/interkeratinocytic	1+

3	Before	Basal, spinous, granular	Diffuse cytoplasmic	2+
	After	Basal, spinous, granular	Diffuse cytoplasmic	1+

4	Before	Basal, spinous, granular	Diffuse cytoplasmic	3+
	After	Basal, spinous, granular	Peripheral/interkeratinocytic	2+

5	Before	Basal, spinous, granular	Diffuse cytoplasmic	2+
	After	Basal, spinous, granular	Peripheral/interkeratinocytic	1+/2+ (only spinous layer 2+)

6	Before	Basal, spinous, granular	Diffuse cytoplasmic	2+
	After	Basal, spinous, granular	Mixt	1+/2+

7	Before	Basal, spinous, granular	Diffuse cytoplasmic	2+
	After	Basal, spinous, granular	Mixt	1+/2+

8	Before	Basal, spinous, granular	Diffuse cytoplasmic	3+
	After	Basal, spinous, granular	Peripheral/interkeratinocytic	1+

9	Before	Basal, spinous, granular	Mixt	1+
	After	Basal	Peripheral/interkeratinocytic	0/1+ (only basal layer 1+)

10	Before	Basal, spinous, granular	Diffuse cytoplasmic	2+
	After	Basal	Peripheral/interkeratinocytic	1+

11	Before	Basal, spinous, granular	Diffuse cytoplasmic	2+
	After	Basal, spinous, granular	Peripheral/interkeratinocytic	2+

12	Before	None		0
	After	None		0

13	Before	Basal, spinous, granular	Diffuse cytoplasmic	2+
	After	Basal, spinous, granular	Peripheral/interkeratinocytic	1+

14	Before	Basal, spinous, granular	Diffuse cytoplasmic	1+
	After	Basal, spinous, granular	Peripheral/interkeratinocytic	1+

15	Before	Basal, spinous, granular	Diffuse cytoplasmic	1+
	After	Basal, spinous, granular	Peripheral/interkeratinocytic	1+
